# Review of the Highly Pathogenic Avian Influenza in Argentina in 2023: Chronicle of Its Emergence and Control in Poultry

**DOI:** 10.3390/pathogens13090810

**Published:** 2024-09-19

**Authors:** Ariel E. Vagnozzi

**Affiliations:** Instituto de Virología e Innovación Tecnológica (IVIT), Centro de Investigación en Ciencias Veterinarias y Agronómicas (CICVyA), Instituto Nacional de Tecnología Agropecuaria (INTA)—Concejo Nacional de Investigaciones Científicas y Técnicas (CONICET), Repetto y de Los Reseros (S/N) Hurlingham, 1686 Buenos Aires, Argentina; vagnozzi.ariel@inta.gob.ar

**Keywords:** highly pathogenic avian influenza, H5 Gs/GD/96 clade 2.3.4.4b, Argentina, stamping out, control, influenza-free status

## Abstract

Highly pathogenic avian influenza (HPAI) is a highly contagious viral disease that represents a significant threat to poultry production worldwide. Variants of the HPAI virus (HPAIV) H5A/Goose/GuangDong/1/96 (H5 Gs/GD/96) lineage have caused five intercontinental epizootic waves, with the most recent, clade 2.3.4.4b, reaching Argentina in February 2023. Initially detected in wild birds, the virus quickly spread to backyard and commercial poultry farms, leading to economic losses, including the loss of influenza-free status (IFS). By March/April 2023 the epidemic had peaked and vaccination was seriously considered. However, the success of strict stamping-out measures dissuaded the National Animal Health Authority (SENASA) from authorizing any vaccine. Suspected cases sharply declined by May, and the last detection in commercial poultry was reported in June. The effective control and potential eradication of HPAIV in Argentina were due to SENASA’s early detection and rapid response, supported by private companies, veterinarians, and other stakeholders. Stamping-out measures have been effective for virus elimination and reduced farm-to-farm transmission; however, as the virus of this clade may remain present in wild birds, the risk of reintroduction into poultry production is high. Therefore, maintaining continuous active surveillance will be crucial for promptly detecting any new HPAIV incursion and taking appropriate action to contain virus dissemination.

## 1. Introduction

Avian influenza (AI) is a highly contagious viral disease that represents a significant threat to poultry production worldwide [1]. The etiologic agent is an alpha influenza virus (*Orthomyxoviridae* family), whose genome comprises eight negative-sense RNA segments encoding both structural and non-structural proteins [2]. Among these, the structural hemagglutinin (HA) and neuraminidase (NA) proteins are crucial for characterization; in fact, the classic AI virus (V) serotype classification relies on the humoral immune response against these proteins [1]. However, over the last two decades, serotyping has been substituted by nucleotide sequence analysis, which provides more detailed and complex information about the numerous AIV variants which allows for expansion of epidemiologic analysis [3]. Sequence analysis also involves describing pathogenicity determinants in different gene segments, as well as identifying reassortments [4].

Although the AI virus (AIV) can infect a wide variety of avian species, birds belonging to the orders *Anseriformes* and *Charadriiformes* are the primary reservoirs, responsible for global dissemination through long-distance migrations [5]. Among wild-bird reservoirs, the virus typically exhibits a highly host-adapted phenotype, replicating in the epithelial cells of the respiratory and gastrointestinal tracts without causing clinical signs [6]. This low pathogenic (LP) virus can be transmitted from wild to domestic birds, resulting in subclinical or mild infections that may eventually progress to a highly pathogenic (HP) disease, which is responsible for massive losses in the poultry industry [7]. It has traditionally been assumed that HPAIV cannot be sustained in wild-bird populations [8]; however, this assumption has been reconsidered since the emergence of the H5 A/Goose/GuangDong/1/96 (H5 Gs/GD/96) lineage [9]. Over the past decades, infections in domestic birds, with subsequent spillovers into wild migratory birds, have been reported, leading to the persistence of variants of the HP H5 Gs/GD/96 lineage. These variants have given rise to five intercontinental waves across Asian, African, European, and American countries, causing substantial losses in poultry and wild birds [9,10]. The most recent of these waves, involving the variant H5N1 Gs/GD/96 clade 2.3.4.4b, spread throughout the South American continent during the summer of 2022–2023 (Southern Hemisphere), raising significant concern in the region [11]. Beyond the direct impact on the poultry industry, there is also a growing concern about the association of these waves with global spillover events involving wild and domestic mammals and their potential ability to transmit to humans, which poses significant implications for public health (reviewed in [12]).

This review provides a brief overview of the HPAIV H5 clade 2.3.4.4b incursion into Argentine territory in 2023, detailing the rapid spread of the virus in commercial and backyard poultry, its economic impact, and the measures taken to control and eradicate the disease within a few months. Most of the information provided here has been gathered from official and public reports.

## 2. Chronology of the Virus Incursion and Epidemic Management

**Figure 1:**
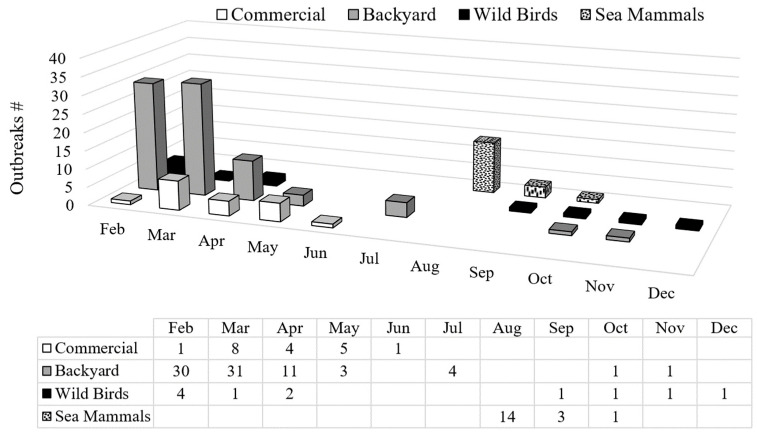
The graph shows the number of cases detected by RRT-PCR reported by SENASA from February to December 2023. The peak of detections in backyard and commercial poultry was observed in February and March. Between August and October, 18 cases in sea mammals were reported. Source: SENASA (https://qliksensebycores.senasa.gob.ar/sense/app/28a22e66-c131-434e-861d-213bab5efc80/sheet/cf22d176-442b-4856-ab37-5210007b06d1/state/analysis, accessed on 13 August 2024).

**Figure 2:**
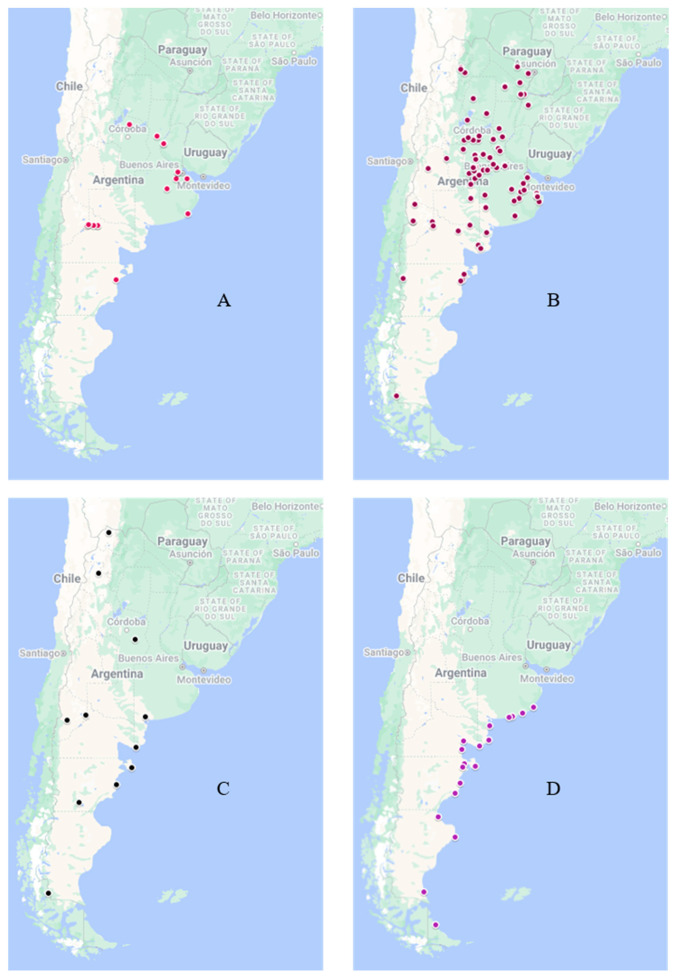
The graph shows the geographical distribution of the outbreaks of HPAIV H5 clade 2.3.4.4b in Argentina. (**A**) Commercial poultry cases, (**B**) backyard poultry cases, (**C**) wild-bird cases, and (**D**) sea mammal cases. More details are in Tables S1–S3. Source: WAHIS WOAH [15,16].

While reports of LPAIV in wild birds have been documented [13,14], no HPAIV was detected in Argentina before 2023. However, on 14 February of that year, HPAIV was reported for the first time, affecting a population of Andean geese (*Oressochen melanopterus*) in a national park in the northern region of the country [15]. Thereafter, the virus rapidly spread to other bird species across the country, including both backyard and commercial poultry [15,16]. The involvement of the latter led to Argentina losing its influenza-free status (IFS), severely impacting the international trade of poultry products. In just 30 days (from 14 February to 15 March), the number of HPAIV detections increased rapidly, and involved 4 outbreaks in wild birds, 59 in backyard birds, and 5 in commercial poultry. The peak of detections occurred shortly after, with the National Veterinary Service (SENASA) reporting 75 epidemic outbreaks by the end of March 2023 (Figure 1). Among these, 5 were in wild birds (with 33 dead birds reported), 61 in backyard birds (resulting in 7819 domestic bird fatalities), and 9 in commercial poultry farms (affecting 1,008,891 birds). Notably, these cases were not concentrated in any particular area but were widely scattered across the territory and affected different provinces, hundreds of kilometers apart (Figure 2). Specifically, the cases detected in the southern part of the country (Neuquén and Río Negro provinces) suggested a possible alternative entry route (of the northern route). Considerable losses were incurred within the first 30 days by several poultry companies, including three breeder and four layer farms, raising alarm among Argentinean producers. Consequently, the National Committee for Poultry Health (CONASA) was convened to discuss potential modifications to epidemic management strategies. Up to that point, disease control had relied on stamping-out procedures and zoning, leading to a serious debate, particularly regarding the use of vaccines. Under experimental conditions, specific vaccines against H5Nx 2.3.4.4b have demonstrated potential in limiting virus spread and preserving animal health; however, they were not entirely effective in inhibiting virus replication [17,18]. This limitation may hinder the prompt eradication of the virus, which is a significant inconvenience for the rapid restoration of the IFS, crucial for a country like Argentina that exports poultry products. While weighing the pros (e.g., limiting virus dissemination) and cons (e.g., failing to achieve complete inhibition of virus replication) of vaccine application, reaching a consensus proved challenging. Thus, SENASA decided not to authorize vaccines unless the situation escalated significantly, opting to continue with the stamping-out strategy, in order to eradicate the HPAIV quickly and recover the IFS as soon as possible. Therefore, biosecurity measures were intensified, and individuals were encouraged to promptly report suspected cases upon observing characteristic signs and lesions. Over the following weeks, throughout April and May, the number of positive cases progressively declined. Notably, the virus never reached the eastern part of Entre Ríos province, where nearly 50% of Argentina’s broiler production is concentrated. This was a significant factor in preventing a more complex scenario.

Finally, the last HPAIV-positive commercial farm was detected on 14 June, and by 7 August, SENASA self-declared Argentina’s commercial poultry free of HPAIV (https://www.woah.org/app/uploads/2023/08/2023-08-argentina-hpai-selfd-sp.pdf, accessed on 13 August 2024).

Additionally, from July to November, the frequency of HPAIV detection in backyard and wild birds progressively decreased (Figure 1, Tables S1 and S2). Simultaneously, several sea mammals found dead along the Argentine coast tested positive for HPAIV (Figure 2D). These detections occurred from August to October, and involved a total of 18 outbreaks (Figure 1). After 20 October, no further cases of infected sea mammals were reported, and the last detection of HPAIV in any animal in Argentina occurred in December (Tables S1–S3).

## 3. Official Measures to Face the HPAIV H5N1 Epidemic

The National Animal Health Authority, SENASA, played a primary role in the prevention, control, and eradication of HPAIV. They prioritized proactive prevention by educating stakeholders (such as farmers, veterinarians, and others who interact with birds) on biosecurity protocols. As the risk of HPAIV introduction increased, these educational efforts intensified, likely contributing to the early detection of suspected cases.

Upon receiving reports of suspected cases, SENASA acted promptly by interdicting affected areas and establishing a sanitary control zone (SCZ) with a prohibition on moving animals and their products. They also conducted diagnostic tests using real-time RT-PCR (RRT-PCR) to confirm or rule out the presence of the virus (Table 1). These actions were implemented not only on commercial farms but also in backyard cases. The rapid establishment of an SCZ was central to curtailing virus dissemination; in fact, 6 out of 18 detections in commercial farms were due to active surveillance within an already established SCZ (Table 2). Once HPAIV was confirmed by RRT-PCR, containment measures (including the euthanasia of affected animals and the proper disposal of carcasses, litter, and feed) were implemented according to established protocols set by law [19]. The contingency measures for HPAIV control encompassed various aspects of containment, zoning for surveillance and movement control, and post-outbreak procedures, as described elsewhere [20]. Table 1 summarizes the main measures. The closure of each outbreak interdiction was achieved at least 28 days following stamping out and cleaning and disinfection of facilities, in accordance with international guidelines, to ensure the virus was eradicated. This process included a study of sentinel birds to confirm the absence of the virus as a prerequisite for repopulating affected poultry establishments. By taking these steps, SENASA aimed to control and reduce the risk of HPAIV dissemination, safeguarding Argentina’s poultry production as well as wildlife. Such proactive measures were crucial in preventing viral spread, protecting both animal and human health.

## 4. Economic Impact

By the end of the 2023 outbreaks, 18 commercial farms had been affected, involving a total of 1,644,744 birds (67,197 breeders, 902,207 laying hens, and 675,340 broilers). The replacement cost for these animals, based on the Argentine market price, was USD 10,276,861.31. Additionally, the cost for the disposal of carcasses, litter, and feed, as well as for cleaning and disinfecting farms and facilities, was estimated at USD 347,168.61, as detailed in Table 2. This resulted in a total cost of USD 10,624,029.92 for the poultry industry, not including downtime costs, such as salaries. Furthermore, the loss of the IFS due to HPAIV in commercial farms led to the cessation of international trade of poultry products. As Argentina is the eighth-largest chicken-meat producer, accounting for 2.3% of global production [21], with almost 10% of its production designated for export, the impact of the HPAIV infection was substantial.

In addition to commercial poultry, a total of 81 outbreaks were reported in backyard poultry, involving 10,472 birds (including hens, geese, ducks, and turkeys) that either died or were culled [15]. The economic losses among backyard holders have not been estimated.

To alleviate these losses, the Argentine national government allocated USD 10,035,936.00 USD to compensate producers affected by the disease [22]. Additionally, certain provincial governments contributed to compensation efforts, although official data on these contributions are unavailable.

Although the compensation was lower than the estimated losses, these funds were instrumental in mitigating damages and, importantly, preventing discouragement in reporting suspected cases.

## 5. Discussion

In 2023, the Argentine poultry industry experienced its first outbreak of HPAIV. The virus, identified as H5 clade 2.3.4.4b, was initially detected in wild birds in a northern province bordering Bolivia [15]. Subsequently, it quickly spread to other locations, although multiple entry routes cannot be ruled out. Within a few days, infections were confirmed in wild birds, backyard poultry, and commercial poultry populations [15,16]. Prompt reporting by individuals in direct or indirect contact with birds (such as veterinarians, park rangers, and farmers) and decisive action by SENASA and the poultry industry were pivotal for controlling the HPAIV outbreak in a short time. Additionally, to facilitate early reporting of suspected cases, the national government offered financial compensation. Control and eradication efforts relied primarily on stamping-out measures. Even though vaccines were seriously considered as a tool to control viral dissemination after infection, the stamping-out procedures, along with zonification and surveillance, have proven sufficient to limit virus spread in Argentina’s situation (where the number of cases was limited).

The rapid establishment of a restricted zone (the SCZ) around suspected cases has been critical in preventing further spread and containing outbreaks. The objective was to prevent potential dissemination and, once the virus was detected, to contain it locally. The aforementioned procedures have been effective in achieving this goal. As a result, the last reported detection of H5N1 clade 2.3.4.4b in Argentine commercial poultry occurred on 14 June 2023 [16]. Argentina officially declared itself free of HPAIV in commercial poultry on 8 August 2023, thereby regaining its IFS status [20]. Since then, only sporadic detections have been reported in backyard flocks and wild birds (not in commercial poultry), with the latest occurrence noted in December 2023 [15]. However, the possibility of future virus detections in wild bird colonies or poultry farms (including backyard holdings) cannot be ruled out, as new infections of clade 2.3.4.4b or other highly pathogenic AIV variants may arise. Therefore, maintaining continuous active surveillance will be crucial for promptly detecting any new incursion of HPAIV and taking appropriate action to contain virus dissemination [23]. However, as noted elsewhere, implementing active surveillance of AIV in wild and domestic birds is challenging, not only due to the technical complexity of the task but also because of insufficient budget allocation [11]. Additionally, various LPAIV variants have been previously reported in wild-bird populations in Argentina [13,14]. The presence of these LPAIV variants in wild birds represents a risk factor due to their potential role as gene donors in reassortment events, which could give rise to new and better-adapted H5 Gs/GD/96 lineage variants [10]. Detecting these variants through a well-instrumented surveillance system could provide valuable information about AIV ecology. On the other hand, hundreds of sea mammals were found dead along the Argentine seashore in August, September, and October [15]. The affected species included Otaria flavescens, Arctophoca australis australis, and Mirounga leonina, across 18 outbreaks (Table S3). These multispecies outbreaks appear to be epidemiologically linked to the infected sea mammals reported earlier in the Pacific Ocean, rather than to the Argentine poultry outbreaks [24,25]. Nevertheless, the sustained transmission among sea mammals, the high pathogenicity of the strain, and the involvement of multiple species quickly raised concern among local sanitary authorities regarding the zoonotic potential of this H5 variant, although no human cases were reported in Argentina.

## 6. Final Remarks

Experience in different countries has shown that dealing with HPAIV infection is challenging. The causes include intricate interactions among hosts, the scale of poultry production and its trade characteristics, the complexity of backyard birds, and the ecology of wild birds, including migration and close contact among different species [26].

The 2023 HPAIV epidemic in Argentina had a considerable economic impact on the poultry industry, not only in terms of the number of affected farms but also due to the loss of external markets, which were not automatically regained after the country quickly restored its IFS.

The success of controlling and eventually eradicating HPAIV in Argentina, based on stamping-out measures, relied on early detection and prompt action facilitated by the unified and coordinated efforts of National Animal Health Authority, in cooperation with private companies, farmers, veterinarians, and other stakeholders. This cohesive response likely prevented the virus from establishing itself in Argentina’s poultry industry.

The rapid control of cases and the consequent drop in the number of outbreaks led SENASA to refrain from authorizing the use of vaccines. Considering the situation, vaccination might have masked cases, impairing the process of eradicating the virus in commercial poultry, and delaying the restoration of IFS.

Although HPAIV has been eradicated from commercial and backyard poultry, the risk of future virus incursions cannot be dismissed. Therefore, the implementation of continuous, coordinated, and long-term active surveillance in strategically chosen locations will be crucial for preparing actions to contain potential viral dissemination in the future.

## Figures and Tables

**Table 1:** Official measures implemented by SENASA to control the HPAIV H5N1 epidemic [20].

Measures	Description
Containment	Prohibition of entry for foreign individuals and vehicles to the affected establishment. Prohibition of entry and exit of birds, avian products, and by-products. Stamping out of affected birds and sanitary disposal of associated materials (carcasses, litter, and feed). Cleaning and disinfection of facilities following stamping out, including a second round of cleaning if necessary. Analysis of bird movements prior to the outbreak.
Zoning	Establishment of a sanitary control zone (SCZ) around the outbreak site, comprising a perifocal zone (with a radius of 3 km) and a surveillance zone (with a radius of 10 km) to monitor and control virus spread. Epidemiological surveillance in the SCZ, with a focus on early detection within the perifocal zone. Movement control within the SCZ, including restrictions on entry and exit for various poultry-related items.
Surveillance	Systematic sampling within the sanitary control zones, including sentinel bird evaluation. Regular molecular diagnostic tests (RRT-PCR) of samples taken in the perifocal and surveillance zones to promptly detect any new cases.

**Table 2:** Commercial farms affected by the HPAIV H5N1 (clade 2.3.4.4b) in Argentina in 2023. The table shows, for each case, the case dates (the date of the official confirmation and finalization of the cleaning and disinfection activities), the location, the type and number of affected birds, and the estimated losses (reposition and cleaning plus disinfection costs). WAHIS ID cases with the same superscript are SCZ-related (as reported in https://wahis.woah.org/#/in-review/4933?reportId=162037&fromPage=event-dashboard-url, accessed on 13 August 2024). The OB_116297 ^B1^ and OB_116802 ^B2^ cases were detected in the SCZs of OB_115602 and OB_115020 (both backyard cases), respectively [16].

WAHIS ID	Case Date (M/D/Y)		Animal Involved		Location		Costs (USD)	
	Confirmation Date	Complete C/D	Type	Number	Province	Department	Bird Reposition	C/D
OB_115219	2/24/2023	5/18/2023	Breeders	32,950	Rio Negro	Gral. Alvear	527,200	9529
OB_114756 ^A^	2/26/2023	5/18/2023	Broilers	225,340	Buenos Aires	Mainque	465,890	41,408
OB_115607	3/3/2023	5/17/2023	Breeders	19,687	Buenos Aires	Mar del Plata	314,992	5898
OB_115608	3/3/2023	3/30/2023	Layers	10,500	Neuquén	Senillosa	90,841	2802
OB_115648 ^A^	3/9/2023	5/18/2023	Broilers	450,000	Rio Negro	Mainque	930,375	82,184
OB_116028	3/11/2023	5/5/2023	Layers	217,000	Rio Negro	Allen	1,877,376	47,902
OB_116297 ^B1^	3/15/2023	3/31/2023	Breeders	14,560	Santa Fe	Colonia Cavour	232,960	4495
OB_116802 ^B2^	3/22/2023	5/18/2023	Layers	2750	Córdoba	El Espinillo	23,792	1109
OB_116803	3/28/2023	4/29/2023	Layers	50,207	Buenos Aires	Lobos	434,366	11,474
OB_117130	4/3/2023	5/30/2023	Layers	407,900	Chubut	Gaiman	3,528,947	89,594
OB_117870 ^C^	4/23/2023	7/3/2023	Layers	34,500	Buenos Aires	Pilar	298,477	8044
OB_117871 ^D^	4/24/2023	6/29/2023	Layers	20,200	Neuquén	Plottier	174,760	4920
OB_118319 ^D^	5/4/2023	7/5/2023	Layers	26,900	Buenos Aires	Plottier	232,725	6384
OB_118317 ^C^	5/5/2023	7/4/2023	Layers	61,000	Neuquén	Pilar	527,742	13,831
OB_118318 ^C^	5/8/2023	6/1/2023	Layers	10,000	Buenos Aires	Pilar	86,515	2693
OB_119020 ^E^	5/19/2023	6/23/2023	Layers	15,350	Entre Ríos	Racedo	132,801	3861
OB_119785 ^E^	5/30/2023	6/30/2023	Layers	17,000	Entre Ríos	Racedo	147,076	4222
OB_120231	6/14/2023	7/9/2023	Layers	28,900	Buenos Aires	La Plata	250,028	6820

Note: M/D/Y: Month, day, year. C/D: Cleaning and disinfection.

## Data Availability

This review has been elaborated with data organized from the official reports of SENASA to the World Animal Health Information System (WAHIS) and the World Organization for Animal Health (WOAH). The source links are https://wahis.woah.org/#/in-review/4908?reportId=159358&fromPage=event-dashboard-url (accessed on 13 August 2024) and https://wahis.woah.org/#/in-review/4933?reportId=162037&fromPage=event-dashboard-url (accessed on 13 August 2024). Those reports (along with other material used) are also available as PDF files in the Supplementary Materials.

## Supplementary Materials

The following supporting information can be downloaded at: https://www.mdpi.com/article/10.3390/pathogens13090810/s1, Table S1: Backyard cases. The data to build this table were collected from SENASA official reports in https://wahis.woah.org/#/in-review/4908?reportId=159358&fromPage=event-dashboard-url (accessed on 13 August 2024); Table S2: The table shows all the wild-bird cases. The information used to elaborate this table was obtained from https://wahis.woah.org/#/in-review/4908?reportId=159358&fromPage=event-dashboard-url (accessed on 13 August2024); Table S3: The table shows the cases of HPAIV H5 reported by SENASA to the WOAH. All the information was obtained from https://wahis.woah.org/#/in-review/4908?reportId=159358&fromPage=event-dashboard-url (accessed on 13 August 2024).

## Abbreviations

HP = highly pathogenic; AIV = avian influenza virus; IFS = influenza-free status; RNA = ribonucleic acid; LP = low pathogenic; SCZ = sanitary control zone; RRT-PCR = real-time reverse transcription polymerase chain reaction; USD = United States dollar.
