# Supplementary material for: Review of the Highly Pathogenic Avian Influenza in Argentina in 2023: Chronicle of Its Emergence and Control in Poultry
Source: Pathogens. 2024 Sep 19;13(9):810. doi: 10.3390/pathogens13090810 (PMC11434679; doi:10.3390/pathogens13090810)
Supplement: Supplementary file 1 [file pathogens-13-00810-s001.zip › Table S1.pdf]

Supplemental material

Table S1: Backyards cases. The data to build this table was collected from SENASA official reports in https://wahis.woah.org/#/in-review/4908?reportId=159358&fromPage=event-dashboard-url (Accessed April 2024)

WAHIS ID	Province	County	Location	Starting date	Closure date	Number of birds
OB_114227	Córdoba	Bellville	-33.64521 , -62.67214	2/16/2023	4/7/2023	288
OB_114228	Salta	Cerrillos	-24.8678 , -65.3921	2/16/2023	3/20/2023	20
OB_114996	Córdoba	Carnerillo	-32.9164 , -63.8423	2/17/2023	4/5/2023	51
OB_115385	Córdoba	Colonia Leguizamón	-34.23677 , -62.97652	2/17/2023	4/14/2023	25
OB_114316	Santa Fe	Villa Cañas	-34.0043 , -61.6175	2/17/2023	4/3/2023	50
OB_115312	Santiago del Estero	Beltran	-27.77411 , -64.07527	2/17/2023	4/14/2023	37
OB_114991	Córdoba	Villa del Rosario	-31.54444 , -63.5195	2/18/2023	3/29/2023	55
OB_114995	Córdoba	Baldisera	-33.1442 , -62.3004	2/18/2023	4/5/2023	150
OB_115006	Córdoba	Rio Primero	-31.2222 , -63.4999	2/19/2023	4/5/2023	57
OB_115010	Córdoba	Del Campillo	-34.5552 , -64.5472	2/19/2023	3/29/2023	126
OB_115311	Buenos Aires	Rauch	-36.66629 , -59.22199	2/20/2023	4/14/2023	120
OB_114999	Buenos Aires	Puan	-37.46847 , -62.75846	2/20/2023	3/29/2023	138
OB_115020	Cordoba	Tulumba	-29.78204 , -64.65244	2/20/2023	4/5/2023	27
OB_115013	Santa Fe	Centeno	-32.28761 , -61.46188	2/20/2023	3/29/2023	50
OB_115316	Neuquén	Las Lajas	-38.54855 , -70.38195	2/22/2023	4/14/2023	91
OB_115021	Río Negro	Choel Choele	-39.39435 , -65.64608	2/22/2023	3/29/2023	132
OB_115026	San Luis	Alto Pencoso	-33.24245 , -66.88304	2/22/2023	4/5/2023	180
OB_115314	Santa Fe	Rufino	-34.25012 , -62.58456	2/22/2023	5/15/2023	119
OB_115024	Buenos Aires	Tres Lomas	-36.4065 , -62.80858	2/23/2023	3/29/2023	380
OB_115027	Buenos Aires	Azul	-36.90228 , -59.8037	2/23/2023	4/5/2023	320
OB_115025	Buenos Aires	San Cayetano	-38.15595 , -59.64585	2/23/2023	3/29/2023	370
OB_115307	Córdoba	Idiazabal	-32.84639 , -62.99188	2/23/2023	3/29/2023	71
OB_115308	Córdoba	Carnerillo	-32.91322 , -63.8743	2/23/2023	4/5/2023	164
OB_115023	Córdoba	Gral. Levalle	-33.92356 , -63.79469	2/23/2023	3/29/2023	100
OB_115408	Buenos Aires	Las Flores	-36.07417 , -59.07298	2/24/2023	4/14/2023	104
OB_115309	Córdoba	Alejandro Roca	-33.28476 , -63.81225	2/24/2023	4/5/2023	114
OB_115306	Córdoba	General Levalle	-33.95114 , -64.01718	2/24/2023	5/3/2023	96
OB_115407	Córdoba	Jovita	-34.33661 , -63.98876	2/24/2023	4/14/2023	100
OB_118940	Córdoba	Jovita	-34.95963 , -63.90554	2/24/2023	5/18/2023	SD
OB_118939	Córdoba	Buchardo	-34.64405 , -63.45809	2/28/2023	5/18/2023	65
OB_115598	Chaco	Avai Terai	-26.6106 , -60.7202	3/1/2023	4/14/2023	161
OB_115315	Neuquén	Añelo	-38.5848 , -68.34685	3/1/2023	4/14/2023	74
OB_115597	Córdoba	La Cautiva	-33.9814 , -64.09017	3/2/2023	4/14/2023	67
OB_115602	Santa Fe	Grutly	-31.28158 , -61.0268	3/4/2023	5/15/2023	74
OB_115649	Buenos Aires	Conesa	-36.47324 , -57.34909	3/7/2023	5/18/2023	479
OB_115650	Córdoba	Toledo	-31.57579 , -64.07424	3/8/2023	5/3/2023	104
OB_115610	La Pampa	Trenel	-35.48092 , -64.32964	3/8/2023	4/21/2023	210
OB_115653	Neuquén	Las Lajas	-38.55657 , -70.37791	3/8/2023	5/3/2023	40
OB_115619	San Luis	Sta. Rosa de Conlara	-32.389 , -65.096	3/8/2023	4/21/2023	60
OB_115651	Santa Fe	Totoras	-32.5628 , -61.2707	3/8/2023	5/18/2023	38
OB_116018	Buenos Aires	Francisco Berra	-35.38236 , -58.83003	3/9/2023	5/3/2023	49
OB_116019	Chaco	Avai Terai	-26.68111 , -60.72653	3/10/2023	4/14/2023	31
OB_116017	Córdoba	Los Morteritos	-31.58601 , -65.0533	3/10/2023	5/3/2023	164
OB_116022	Buenos Aires	Conesa	-36.27949 , -57.39065	3/11/2023	5/18/2023	139
OB_116021	Neuquén	Plottier	-38.96685 , -68.2701	3/11/2023	5/3/2023	7
OB_116020	Santa Fe	Zenon Pereira	-31.53552 , -61.96289	3/11/2023	5/15/2023	134
OB_116023	Buenos Aires	Conesa	-36.46904 , -57.43139	3/13/2023	5/18/2023	42
OB_116026	Buenos Aires	Gral Madariaga	-36.97864 , -57.15243	3/13/2023	5/15/2023	68
OB_116024	Buenos Aires	Pedro Luro	-39.48708 , -62.68335	3/13/2023	5/15/2023	141
OB_116025	Neuquén	Las Lajas	-38.556134 , -70.37928	3/13/2023	5/3/2023	50

OB_116027	La Pampa	Toay	-36.69454 , -64.31648	3/16/2023	5/3/2023	145
OB_116295	Buenos Aires	Conesa	-36.52865 , -57.34021	3/17/2023	5/18/2023	42
OB_116293	Río Negro	Guardia Mitre	-40.52014 , -63.54515	3/17/2023	5/15/2023	241
OB_116294	Chubut	Rawson	-43.26625 , -65.17823	3/20/2023	5/15/2023	65
OB_116494	Neuquén	Chapua	-37.181053 , -70.24798	3/20/2023	5/3/2023	100
OB_116491	Neuquén	Las Lajas	-38.55845 , -70.37815	3/21/2023	3/24/2023	56
OB_116796	Buenos Aires	General Belgrano	-35.95611 , -58.73683	3/24/2023	5/19/2023	585
OB_116797	Santa Fe	Venado Tuerto	-33.73278 , -61.92956	3/27/2023	5/3/2023	89
OB_116996	Chubut	Trevelin	-43.05213 , -71.45945	3/30/2023	5/3/2023	484
OB_117129	Santa Fe	Capivara	-30.502 , -61.34395	3/30/2023	6/16/2023	186
OB_116998	Corrientes	San Cosme	-27.39907 , -58.67021	3/31/2023	5/3/2023	94
OB_117314	Río Negro	Río Colorado	-38.888405 , -64.413895	4/2/2023	6/16/2023	103
OB_117405	Formosa	Colonia Alba	-26.2965 , -59.1817	4/9/2023	5/15/2023	180
OB_117680	Chubut	Puerto Madryn	-42.7996 , -65.08174	4/11/2023	6/16/2023	43
OB_117681	Río Negro	Viedma	-40.76601 , -63.29278	4/16/2023	6/8/2023	194
OB_117873	Santa Cruz	Rio Turbio	-51.51924 , -72.18606	4/17/2023	6/2/2023	141
OB_117875	Corrientes	Tatacua	-28.36806 , -58.25909	4/18/2023	6/16/2023	131
OB_117876	Chubut	Trevelin	-43.13071 , -71.43777	4/19/2023	6/16/2023	152
OB_117874	Mendoza	San Carlos	-34.10899 , -68.778496	4/19/2023	5/24/2023	53
OB_117877	Buenos Aires	Almirante Brown	-34.85651 , -58.345436	4/20/2023	5/24/2023	159
OB_117878	Buenos Aires	Pergamino	-33.89367 , -60.69711	4/21/2023	6/8/2023	280
OB_118113	Chaco	Puerto Triol	-27.41668 , -59.02865	4/30/2023	6/16/2023	11
OB_118323	Formosa	Riacho He He	-25.36837 , -58.265809	5/2/2023	6/16/2023	233
OB_118324	Chubut	Trelew	-43.30236 , -65.3495	5/8/2023	6/16/2023	40
OB_118938	Chaco	Puerto Triol	-27.40108 , -59.07218	5/10/2023	6/16/2023	8
OB_121842	Formosa	Villa Gral.Guemes	-24.75241 , -59.44938	7/5/2023	8/7/2023	112
OB_137179	Salta	Rio Piedras	-25.3118 , -64.9089	7/26/2023	8/30/2023	76
OB_137191	Córdoba	Cerro Blanco	-31.34564 , -64.63783	7/27/2023	9/20/2023	415
OB_137192	Santiago del Estero	Aguirre	-29.14311 , -62.6803	7/31/2023	9/1/2023	76
OB_137193	Buenos Aires	Gral. Alvear	-35.87469 , -60.05989	10/17/2023	11/21/2023	80
OB_137228	Salta	Vaqueros	-24.6914 , -65.423677	11/15/2023	12/17/2023	166
