# Supplementary material for: Review of the Highly Pathogenic Avian Influenza in Argentina in 2023: Chronicle of Its Emergence and Control in Poultry
Source: Pathogens. 2024 Sep 19;13(9):810. doi: 10.3390/pathogens13090810 (PMC11434679; doi:10.3390/pathogens13090810)
Supplement: Supplementary file 1 [file pathogens-13-00810-s001.zip › Table S2.pdf]

Supplemental material

Table S2: The table shows all the wild birds' cases. The information used to elaborate this table were obtained from https://wahis.woah.org/#/in-review/4908?reportId=159358&fromPage=event-dashboard-url (Accessed April 2024)

WAHIS ID	Province	County	Location	Starting date	Closure date	Number of birds	Species
OB_114176	Jujuy	Laguna de los Pozuelos	-22.4027 , -65.9707	2/14/2023	3/11/2023	9	Oressochen melanopterus
OB_114264	Córdoba	Las Mojarras	-32.2696 , -63.2463	2/15/2023	3/16/2023	2	Anatidae spp
OB_114997	Neuquén	Laguna Blanca	-39.052883 , -70.349562	2/18/2023	3/19/2023	4	Rallidae spp
OB_115313	Buenos Aires	Bahia Blanca	-38.78775 , -62.26203	2/28/2023	3/29/2023	1	*Anatidae spp*
OB_116499	Neuquén	San Patricio del Chañar	-38.59764 , -68.390522	3/21/2023	5/3/2023	17	*Cygnus melancoryphus*
OB_117684	Chubut	Sarmiento	-45.3259 , -69.0711	4/17/2023	5/19/2023	8	*Cygnus melancoryphus*
OB_117872	Santa Cruz	28 de noviembre	-51.57301 , -72.20948	4/17/2023	5/19/2023	1	Oressochen melanopterus
OB_137351	Río Negro	Punta Bermeja	-41.15491 , -63.1613	9/29/2023	10/26/2023	2	*Gelochelidon nilotica*
OB_137249	Chubut	Puerto Madryn	-42.76682 , -63.63556	10/20/2023	11/16/2023	200	*Sterna hirundinacea*
						20	*Larus dominicanus*
						2	*Leucocarbo atriceps albiventer*
OB_137262	Catamarca	El Peñon	-26.2474 , -67.05807	10/30/2023	11/26/2023	10	*Phoenicopterus chilensis*
OB_137385	Chubut	Punta Tombo	-44.0453 , -65.2363	12/24/2023	1/20/2024	1	*Sterna hirundinacea*
