# Supplementary material for: Review of the Highly Pathogenic Avian Influenza in Argentina in 2023: Chronicle of Its Emergence and Control in Poultry
Source: Pathogens. 2024 Sep 19;13(9):810. doi: 10.3390/pathogens13090810 (PMC11434679; doi:10.3390/pathogens13090810)
Supplement: Supplementary file 1 [file pathogens-13-00810-s001.zip › Table S3.pdf]

Supplemental material

Table S3: The table shows the cases of HPAIV H5 reported for SENASA to the WOAH. All the information were obtained from https://wahis.woah.org/#/in-review/4908?reportId=159358&fromPage=event-dashboard-url (Accessed April 2024)

WAHIS ID	Province	County	Location	Detection date	Number of animals	Species
OB_124321	Tierra del Fuego	Rio Grande	-53.71557 , -67.80045	8/8/2023	26	*Otaria flavescens*
OB_124322	Rio Negro	Loberia	-41.15491 , -63.1613	8/11/2023	104	*Otaria flavescens*
OB_124358	Santa Cruz	Puerto Loyola	-51.60741 , -69.01103	8/15/2023	2	*Otaria flavescens*
OB_124359	Buenos Aires	Necochea	-38.58033 , -58.7029	8/21/2023	133	*Otaria flavescens*
					6	*Arctophoca australis australis*
OB_124585	Buenos Aires	Mar del Plata	-38.0512 , -57.5352	8/22/2023	214	*Otaria flavescens*
					2	*Arctophoca australis australis*
OB_124584	Chubut	Piramides	-42.5803 , -64.7913	8/22/2023	4	*Otaria flavescens*
OB_124756	Buenos Aires	Orense	-38.8 , -59.73	8/23/2023		*Otaria flavescens*
OB_124586	Buenos Aires	Bahia San Blas	-40.69639 , -62.23489	8/24/2023	15	*Otaria flavescens*
OB_124588	Buenos Aires	Claromeco	-38.863 , -60.09669	8/26/2023	11	*Otaria flavescens*
					1	*Arctophoca australis australis*
OB_124589	Buenos Aires	Mayor Buratovich	-39.59722 , -62.10915	8/26/2023	11	*Otaria flavescens*
OB_124587	Rio Negro	Punta Villarino	-40.8 , -64.88746	8/26/2023	1	*Otaria flavescens*
					3	*Arctophoca australis australis*
OB_124590	Santa Cruz	Caleta Olivia	-46.469051 , -67.496	8/29/2023	3	*Otaria flavescens*
OB_124757	Chubut	Puerto Madryn	-42.7933 , -64.965	8/30/2023	1	*Otaria flavescens*
OB_124758	Rio Negro	Sierra Grande	-41.426027 , -65.044308	8/30/2023	8	*Otaria flavescens*
OB_124759	Santa Cruz	Puerto Deseado	-47.91 , -65.72	9/3/2023	10	*Otaria flavescens*
OB_124760	Chubut	Camarones	-44.7502 , -65.7155	9/3/2023	84	*Otaria flavescens*
OB_124986	Chubut	Punta Tombo	-44.044823 , -65.22235	9/7/2023	3	*Otaria flavescens*
					1	*Mirounga leonina*
OB_126883	Chubut	Punta Delgada	-42.76682 , -63.63556	10/20/2023	300	*Mirounga leonina*
